# Sex difference in race performance and age of peak performance in the Ironman Triathlon World Championship from 1983 to 2012

**DOI:** 10.1186/2046-7648-1-15

**Published:** 2012-12-14

**Authors:** Christoph A Rüst, Beat Knechtle, Thomas Rosemann, Romuald Lepers

**Affiliations:** 1 Institute of General Practice and Health Services Research, University of Zurich, Zurich, 8091, Switzerland; 2 Gesundheitszentrum St. Gallen, St. Gallen, 9011, Switzerland; 3 INSERM U1093, Faculty of Sport Sciences, University of Burgundy, Dijon CEDEX, 21078, France

**Keywords:** Swimming, Cycling, Running, Ultra-endurance

## Abstract

**Background:**

The fastest Ironman race times in ‘Ironman Hawaii’ were achieved in very recent years. This study investigated the change in sex difference in both race performance and the age of peak performance across years in the top ten athletes for split disciplines and overall race time in the ‘Ironman Hawaii’ between 1983 and 2012.

**Methods:**

Changes in split times, overall race times, and age of athletes across years for the top ten overall and the fastest swimmers, cyclists, and runners were investigated using regression analyses and analyses of variance.

**Results:**

Between 1983 and 2012, the overall top ten men and women finishers improved their swimming (only men), cycling, running, and overall race times. The sex difference in overall race time decreased significantly (*p* = 0.01) from 15.2% to 11.3% across time. For the split disciplines, the sex difference remained unchanged (*p* > 0.05) for swimming (12.5 ± 3.7%) and cycling (12.5 ± 2.7%) but decreased for running from 13.5 ± 8.1% to 7.3 ± 2.9% (*p* = 0.03). The time performance of the top ten swimmers remained stable (*p* > 0.05), while those of the top ten cyclists and top ten runners improved (*p* < 0.01). The sex difference in performance remained unchanged (*p* > 0.05) in swimming (8.0 ± 2.4%), cycling (12.7 ± 1.8%), and running (15.2 ± 3.0%). Between 1983 and 2012, the age of the overall top ten finishers and the fastest swimmers, cyclists, and runners increased across years for both women and men (*p* < 0.01).

**Conclusions:**

To summarize, for the overall top ten finishers, the sex difference decreased across years for overall race time and running, but not for swimming and cycling. For the top ten per discipline, the sex difference in performance remained unchanged. The athletes improved their performances across years although the age of peak performance increased.

## Background

The sex difference in endurance performance has been investigated for swimming [1-4], running [5-8], and multi-sports disciplines such as triathlon [9-13] and duathlon [14]. While some authors argued that women might be able to reach men's endurance performance [5,15,16], others suggested that the sex difference is of biological origin and that women would not be able to approach men's performances [7,17,18]. The sex difference in performance is mainly explained by differences in physiological and anthropometric characteristics. For example, men have a higher aerobic capacity [19] and greater muscle strength [7,17] compared to women.

Previous studies investigated the changes of the sex difference in ultra-endurance performance for single [1-3] and multi-sports disciplines [9,10,13]. Long-distance triathlon such as the Ironman distance covering 3.8-km swimming, 180-km cycling, and 42.195-km running is an ideal example of an ultra-endurance event defined as an event lasting more than 6 h [20]. The ‘Ironman Hawaii’ triathlon with the first edition in 1978 is the premier event in the field of long-distance triathlon [10]. The fastest race times in Ironman Hawaii were recently achieved for both men and women. The current Ironman Hawaii course record for men was set in 2011 by Craig Alexander (Australia) winning the race within 8 h 3 min and 56 s. Chrissie Wellington (Great Britain) set the women's course record in 2009 with a winning time of 8 h 54 min and 2 s (http://ironman.com). The sex difference between these two race times was 10.6%. However, the actual fastest race times for the Ironman distance were set in 2011 in the ‘Challenge Roth’, Germany for men by Andreas Raelert (Germany) winning in 7 h 41 min and 33 s. For women, Chrissie Wellington (Great Britain) finished after 8 h 18 min and 13 s with a corresponding sex difference of 8%.

Apart from the sex difference in performance, the age of peak performance is of interest for ultra-endurance athletes. Previous studies examined the age of peak performance in endurance and ultra-endurance athletes such as marathoners and ultra-marathoners. For elite marathoners, women were older (29.8 ± 4.2 years) than men (28.9 ± 3.8 years) [21]. For 161-km ultra-marathoners, Hoffman and Wegelin [22] reported mean ages of 44.4 ± 9.0 years for men and 41.7 ± 8.0 years for women, respectively. For 100-km ultra-marathoners, the fastest 100-km running times were observed for the age comprised between 30 and 49 years for men and between 30 and 54 years for women [23]. It has been shown that the performance of master triathletes—defined as athletes older than 35 years—improved across years in both Ironman Hawaii [13] and in a qualifier for Ironman Hawaii [24]. However, no study investigated the sex difference in the age of peak performance in long-distance triathletes such as Ironman triathletes.

Lepers [10] investigated the change in sex difference in performance for the overall top ten triathletes competing in the Ironman Hawaii triathlon between 1988 and 2007. Over this period, the sex differences in race time for swimming, cycling, running, and overall race time for the top ten overall triathletes were 9.8 ± 2.9%, 12.7 ± 2.0%, 13.3 ± 3.1%, and 12.6 ± 1.3%, respectively. During the 1988–2007 period, the sex difference remained stable and practically identical for swimming (+0.1% per decade), increased a little for cycling (+0.8% per decade), and decreased more for running (−2.8% per decade). The sex difference in overall race time also remained stable in the last two decades (−0.5% per decade). However, Lepers [10] focused on the overall top ten finishers, and it would be interesting to examine if the sex difference in performance for the best swimmers, the best cyclists, and the best runners at the Ironman Hawaii triathlon follows the same trends as has been reported for the split times of the overall top ten triathletes. In addition, the fastest race times in Ironman Hawaii were achieved after 2007.

Since some authors argued that women would be able to outrun men in ultra-endurance distances [5,16], the first aim of the study was to investigate whether the gap between men and women for the best swimmers, the best cyclists, the best runners, and the best overall performers decreased across years in the Ironman Hawaii triathlon when investigating a 29-year period of time from 1983 to 2012. The second aim of the study was to investigate the change in the age of the best swimmers, the best cyclists, the best runners, and the best overall performers at the Ironman Hawaii triathlon within the same period of time.

## Methods

The present study was approved by the Institutional Review Board of St. Gallen, Switzerland with waiver of the requirement for informed consent given that the study involved the analysis of publicly available data. Participation, performance, and age trends in the Ironman Hawaii were analyzed between 1983 and 2012. The data set from this study was obtained from the race website of Ironman Hawaii triathlon (http://www.ironmanworldchampionship.com). Data before 1983 were not considered in this study due to incomplete data regarding the age of the athletes between the first race in 1978 and 1982.

Data from 41,463 athletes who successfully finished Ironman Hawaii between 1983 and 2012 were available and could be included into the analysis. For this time period of 30 years, the top ten men and the top ten women overall finishers and the ten fastest men and the ten fastest women triathletes in each of the three disciplines were identified (*n* = 30 years × 4 times × 2 sexes × 10 triathletes = 2, 400).

To calculate the sex difference in performance, we used the following formula: (performance in women − performance in men) / performance in men × 100. The sex difference was calculated for the two individuals of different sexes reaching the same ranking within the same year and discipline (e.g., 1995 (total race time), No. 1 man and No. 1 woman; 1996 (cycling split time) No. 5 man and No. 5 woman. Afterwards, the sex differences of the overall top ten finishers and the ten fastest performers of each discipline were pooled within the same year. All sex differences were transformed to absolute values before analysis.

### Statistical analysis

In order to increase the reliability of the data analyses, each set of data was tested for normal distribution as well as for homogeneity of variances prior to statistical analyses. Normal distribution was tested using D'Agostino and Pearson omnibus normality test, and homogeneity of variances was tested using Levene's test in case of two groups and with a Bartlett's test in case of more than two groups. To find significant changes in performance, sex difference, and age across years, linear regressions were used. To find differences in age between female and male triathletes in the different disciplines, a two-way analysis of variance (ANOVA) with sex × discipline was performed with subsequent Bonferroni post-hoc analysis. In addition, the interaction between split discipline and time (years) on sex difference was determined using a two-way ANOVA. Differences between two groups were analyzed using Student's *t* test in case of normal distributed data, with Welch's correction in case of different variances between the two analyzed groups and with Mann–Whitney test in case of not normal distributed data. Differences between multiple groups were determined using one-way-ANOVA with subsequent Tukey-Kramer post-hoc analysis. Statistical analyses were performed using IBM SPSS Statistics (Version 19, IBM SPSS, Chicago, IL, USA) and GraphPad Prism (Version 5, GraphPad Software, La Jolla, CA, USA). Significance was accepted at *p* < 0.05 (two-sided for *t* tests). Data in the text are given as mean ± standard deviation.

## Results

### Participation trends

**Figure 1:**
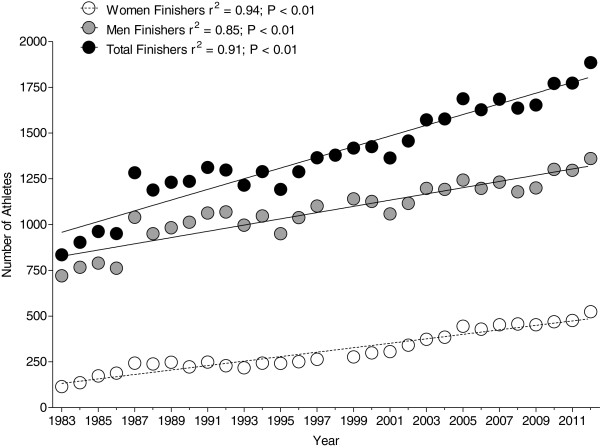
Change in participation across the years for both men and women at the Ironman Hawaii.

During the investigated period from 1983 to 2012, the number of successful finishers increased for both women and men (Figure 1). The number of male finishers increased from 720 (1983) to 1,362 (2012) by 189%; the number of female finishers increased from 115 (1983) to 524 (2012) by 455%.

### Sex difference in performance

#### The overall top ten finishers

**Figure 2:**
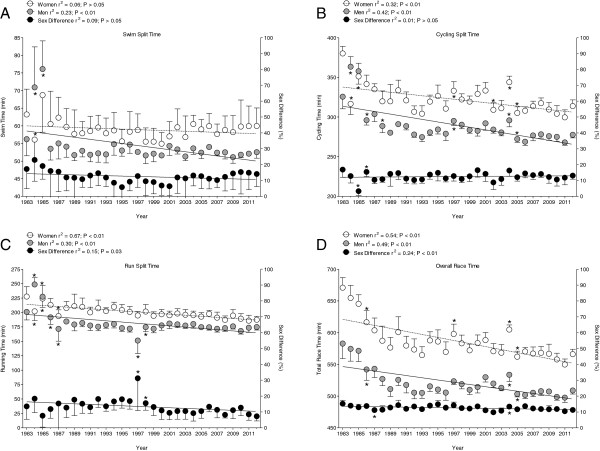
**Change in swimming, cycling, and running split times and overall race times.** (**A**) Swimming split times, (**B**) cycling split times, (**C**) running split times, and (**D**) overall race times of the overall top ten male and the overall top ten female finishers at the Ironman Hawaii. An asterisk indicates values which are significantly different from the previous one.

During the 1983–2012 period, the swim time remained unchanged (*p* > 0.05) for women at 58.9 ± 2.7 min but decreased significantly (*p* < 0.01) for men from 56.2 ± 4.7 min to 52.5 ± 1.7 min (−7.6%) (Figure 2A). The sex difference in swimming performance remained stable at 12.5 ± 3.7% (*p* > 0.05). The cycle time decreased significantly (*p* < 0.01) from 380.3 ± 8.9 min to 313.9 ± 7.8 min (−17.5%) for women and from 325.7 ± 15.3 min to 277.7 ± 3.9 min (−14.7%) for men (Figure 2B). The sex difference in cycling performance remained unchanged at 12.5 ± 2.7% (*p* > 0.05). For the marathon, the running time decreased significantly (*p* < 0.01) from 227.4 ± 17.4 min to 187.4 ± 5.7 min (−17.6%) for women and from 200.9 ± 14.0 min to 174.7 ± 5.3 min (−13.0%) for men (Figure 2C). The sex difference in running performance decreased significantly (*p* = 0.03) from 13.5 ± 8.1% to 7.3 ± 2.9%.The overall race time decreased significantly (*p* < 0.01) from 671 ± 16 min to 566 ± 8 min (−15.6%) for women and from 583 ± 24 min to 509 ± 6 min (−12.7%) for men (Figure 2D). The sex difference in overall race time decreased significantly (*p* < 0.01) from 15.2% to 11.3% during the studied period.

#### The top ten fastest performances in each discipline

**Figure 3:**
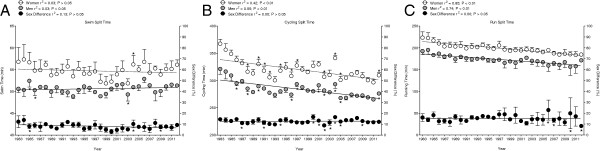
**Change in performance of the ten fastest swimmers, cyclists, and runners.** (**A**) The ten fastest swimmers, (**B**) the ten fastest cyclists, and (**C**) the ten fastest runners at the Ironman Hawaii. An asterisk indicates values which are significantly different from the previous one.

The split performances of the top ten swimmers, the top ten cyclists, and the top ten runners were analyzed separately. For swimming, the ten fastest female and the ten fastest male swimmers showed no significant improvements (*p* > 0.05) in their split times (Figure 3A). Women swam 57 ± 3 min in 1983 and 56 ± 2 min in 2012, while men swam 51 ± 1 min in 1983 and 51 ± 1 min in 2012, respectively. The sex difference in swim performance remained unchanged (*p* > 0.05) at 8.0 ± 2.4%. The ten fastest female and the ten fastest male cyclists improved their bike split times significantly (*p* < 0.01) across time (Figure 3B). Women reduced their split times from 368 ± 8 min to 310 ± 5 min by 16%, and men improved their bike split times by 14% from 321 ± 10 min to 276 ± 3 min. However, the sex difference in cycling performance remained unchanged (*p* > 0.05) at 12.7 ± 1.8%. The ten fastest female runners improved significantly (*p* < 0.01) their marathon split time from 223 ± 13 min to 184 ± 3 min by 17%. Similarly, the ten fastest male marathoners improved significantly (*p* < 0.01) their marathon time from 192 ± 8 min to 171 ± 3 min by 11% (Figure 3C). The sex difference in running performance remained unchanged at 15.2 ± 3.0%.

### Difference between overall top ten finishers and the fastest swimmers, cyclists and runners

During the studied period, the averaged swim split times of the top ten swimmers (women 54.7 ± 1.4 min and men 50.6 ± 1.0 min, respectively) were significantly (*p* < 0.001) faster compared to the swim split times of the overall top ten finishers (women 58.9 ± 2.7 min and men 54.3 ± 5.4 min, respectively). The sex difference in swimming performance was significantly (*p* < 0.001) lower for the top ten swimmers compared to the overall top ten finishers (8.0 ± 2.4% vs. 12.5 ± 3.7%). The average cycle times of the top ten cyclists and the overall top ten finishers were not different for both women and men (women 320.1 ± 17.2 min vs. 322.0 ± 17.0 min; men 284.2 ± 13.8 min vs. 289.7 ± 22.5 min, respectively). The sex difference in cycling performance was not significantly different (*p* > 0.05) between the top ten cyclists and the overall top ten finishers (12.7 ± 1.8% vs. 12.5 ± 2.7%, respectively). The average running times of the top ten runners and the overall top ten finishers were not different for both women (*p* > 0.05) and men (*p* = 0.02) (women 198.6 ± 10.5 min vs. 200.3 ± 10.1 min; men 172.8 ± 9.3 min vs. 180.6 ± 17.7 min, respectively). The sex difference in running performance was significantly (*p* = 0.012) different between the top ten runners and the overall top ten finishers (15.2 ± 3.0% vs. 13.4 ± 4.7%, respectively).

### The age of the fastest athletes

**Figure 4:**
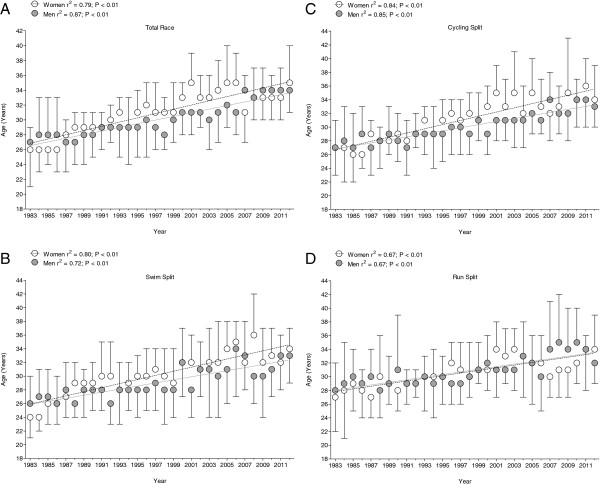
**Changes in age across years for overall top ten male–female finishers, swimmers, cyclists, and runners.** (**A**) The overall top ten male and the overall top ten female finishers, (**B**) the ten fastest swimmers, (**C**) the ten fastest cyclists, and (**D**) the ten fastest runners at the Ironman Hawaii.

Between 1983 and 2012, the age of the overall top ten finishers increased significantly (*p* < 0.01) from 26 ± 5 years to 35 ± 5 years for women and from 27 ± 2 years to 34 ± 3 years for men, respectively (Figure 4A). The mean age of the top ten swimmers increased significantly (*p* < 0.01) for both women and men (women from 24 ± 3 years to 34 ± 3 years; men from 26 ± 4 years to 33 ± 4 years, respectively) (Figure 4B). Similarly, the mean age of the top ten cyclists increased significantly (*p* < 0.01) for both women and men (women from 27 ± 4 years to 34 ± 5 years; men from 27 ± 4 years to 33 ± 3 years, respectively) (Figure 4C). The mean age of the top ten runners increased significantly (*p* < 0.01) for both women and men (women from 27 ± 5 years to 34 ± 5 years; men from 28 ± 4 years to 32 ± 3 years, respectively) (Figure 4D). ANOVA showed no sex difference in the age for the overall top ten finishers, the ten fastest swimmers, the ten fastest cyclists, and the ten fastest runners.

### Interaction between discipline and time (years) on sex difference

The analysis of the sex difference depending on the split discipline (i.e., swimming, cycling, and running) for the top ten athletes per split discipline and with time (i.e. years) showed a highly significant (*F* = 5.5; *p* < 0.01) interaction between discipline and time, accounting for 15.8% of the total variance. Discipline accounted for 35.9% (*F* = 363.6; *p* < 0.01) and time for 8.4% (*F* = 5.9; *p* < 0.01) of the total variance. The same analysis regarding the sex difference in the different disciplines for the overall top ten athletes finishers showed a highly significant (*F* = 3.0; *p* < 0.01) interaction between discipline and time, accounting for 16.3% of the total variance, whereas time accounted for 6.8% (*F* = 2.5; *p* < 0.01) of the total variance, and discipline as a single factor showed no significant impact on the total variance. Over the 1983–2012 period, the mean sex difference for the overall top ten finishers in swimming (12.5 ± 3.7%) was similar (*p* > 0.05) for cycling (12.5 ± 2.7%) and running (13.4 ± 4.7%). For the top ten athletes of each discipline, the mean sex difference in swimming (8.0 ± 2.4%) was different (*p* < 0.05) for cycling (12.7 ± 1.8%) and running (15.2 ± 3.0%).

## Discussion

We investigated, firstly, the change in sex difference performance for the overall top ten finishers and the top ten swimmers, the top ten cyclists, and the top ten runners in Ironman Hawaii between 1983 and 2012, and, secondly, the change in the age of these triathletes across time.

### Sex difference in performance for top ten overall

The present findings showed for the overall top ten finishers that overall race time and the split times in cycling and running decreased for both women and men, whereas the split time in swimming decreased for men, but not for women. However, the sex difference in performance remained unchanged for the split disciplines but decreased from 15.2% to 11.3% for overall race time. It seems that women were able to reduce the sex difference in overall performance in the Ironman Hawaii during the last years. The overall top ten women improved their overall race time by 15.6%, the overall top ten men only by 12.7%.

These results are different from the findings of Lepers [10] investigating the time period between 1981 and 2007 in the Ironman Hawaii. During that period, the sex difference in performance remained stable for swimming, increased for cycling, decreased for running, and remained unchanged for overall race time. These different findings are most probably due to the longer time period investigated in the present study. While Lepers investigated the time period 1981–2007 [10], we investigated the time period 1983–2012. Lepers [10] showed a steep decrease in overall race times between 1981 and 1988. However, overall race times changed not very much between 2007 and 2012 (Figure 2D).

In 2012, the sex difference in overall performance was at 11.3%. A sex difference of approximately 10–12% in Ironman triathletes seems to be of biological origin. Success in endurance performance is mainly determined by aerobic capacity and muscular strength. Because men possess a larger aerobic capacity and a greater muscular strength compared to women, the gap in endurance performances between women and men is unlikely to narrow naturally [7]. Thibault et al. [25] reported a dominance of men when investigating the sex differences in different sports disciplines such as swimming, athletics, track cycling, weightlifting, and speed skating. They determined sex as a major variable of athletic performance by comparing the best performances of women and men in different sports. In every discipline, male performances were significantly better than female performances with a sex difference of 10.0 ± 2.9%.

The sex difference in performance of approximately 10–12% between female and male endurance athletes might be explained by physiological differences such as maximum oxygen uptake (VO_2_max) [19] and anthropometric characteristics such as skeletal muscle mass and body fat [26,27]. Endurance-trained male triathletes have a VO_2_max of 61.3 ml·min^−1^·kg^−1^ compared to female triathletes with a VO_2_max of 52.8 ml·min^−1^·kg^−1^[18] and a corresponding sex difference of 8.5 ml·min^−1^·kg^−1^ (13.9%). It has been shown that male ultra-endurance athletes had a higher skeletal muscle mass than female ultra-endurance athletes [26-29]. Regarding ultra-runners, male ultra-runners with approximately 38 kg of skeletal muscle mass [30] had an approximately 28% higher muscle mass compared to female ultra-runners with approximately 27.4 kg of muscle mass [31]. The higher skeletal muscle mass in men might be an important variable of performance for ultra-endurance athletes and may explain the sex difference. Also for Ironman triathletes, differences in anthropometric characteristics between women and men have been reported. Male Ironman triathletes with approximately 41 kg of skeletal muscle mass had an approximately 32% higher skeletal muscle mass compared to female Ironman triathletes with approximately 28 kg of skeletal muscle mass [29]. The higher skeletal muscle mass may help men to sustain a higher speed for a longer time during an ultra-endurance race compared to women. When women and men were compared regarding their cycling performance, the main factor accounting for sex differences in peak and mean power output during cycling was skeletal muscle mass of the lower extremities [32]. Women with a lower lean leg volume developed a lower peak power compared to men [33]. Apart from skeletal muscle mass, also body fat might be responsible for the sex difference in performance. Body fat has been shown as an important predictor variable for male Ironman triathletes [26]. The lower body fat in men might enhance their endurance performance. Male triathletes with 19.1% body fat have a 29% lower body fat compared to female triathletes with 26.9% body fat [19].

### The sex difference in performance for split disciplines

For the ten fastest performers in each discipline, the cycling and running times improved across years, but not the swim times. The ten fastest cyclists and the ten fastest runners became faster across time, but the sex difference in performance remained unchanged. The sex difference was at 8.0 ± 2.4% in swimming, 12.7 ± 1.8% in cycling, and 15.2 ± 3.0% in running. The low sex difference in swim performance of 8.0 ± 2.4% is explained by the non-significant decrease in swim times of approximately 1 min for women and the non-significant change in swim times for men. Women swam 57 ± 3 min in 1983 and 56 ± 2 min in 2012, while men swam 51 ± 1 min in 1983 and 51 ± 1 min in 2012.

We assume that Ironman triathletes invested more time in the preparation to improve in those split disciplines with more importance for the race. The 3.8-km swim split accounts for only 1.7% of the total distance, whereas the 180-km cycling accounts for 79.6% and the marathon for 18.7% of the total race distance of 226 km. It has been shown for ultra-triathletes that both the cycling and the run splits were associated with overall race performance, but not the swim split [34].

### Difference between the performances of the overall top ten finishers and the performances of the ten fastest swimmers, the ten fastest cyclists, and the ten fastest runners

When the performances between the overall top ten finishers and the performances of the ten fastest swimmers, the ten fastest cyclists, and the ten fastest runners were compared, both the ten fastest male and the ten fastest female swimmers swam faster than the overall ten fastest finishers. For cycling, the overall top ten finishers and the ten fastest cyclists achieved the same split times for both sexes. For running, the overall top ten finishers and the ten fastest marathoners achieved the same split times in women, but not in men. A potential explanation for the finding that the ten fastest male and the ten fastest female swimmers swam faster than the overall ten fastest finishers could be the ‘history’ of the athlete. It has been reported that many triathletes have previously been competitive swimmers [35]. For recreational Ironman triathletes competing in ‘Ironman Lanzarote’, 28% of the athletes had a background as a runner, 14% as a swimmer, and 13% as a cyclist [36]. For elite Ironman triathletes competing in Ironman Hawaii, however, the history of the athletes has not been investigated.

A more likely explanation for the better performance in the top ten swimmers compared to the overall top ten finishers could be the fact that wetsuits are prohibited in Ironman Hawaii. Wearing a wetsuit leads to a significantly lower swimming cadence (−14%), a significantly lower heart rate (−11%), and significantly lower lactate values (−47%) compared to swimming without a wetsuit [37]. Moreover, cycling efficiency was significantly higher (+12.1%) after swimming with a wetsuit compared to swimming without a wetsuit. In an Ironman triathlon where athletes can wear a wetsuit in the swim split, athletes without the background as a swimmer may profit from wearing a wetsuit and achieve faster swim times.

However, in Ironman Hawaii, athletes with a background as a swimmer may swim fast in open water independent of whether they wear a wetsuit or not since they are used as swimmers to swimming fast in contrast to a weak swimmer. It has been shown that wearing a wet suit improves swim performance more in inefficient swimmers with low buoyancy when swimming at low speeds [38].

### The age of the fastest Ironman triathletes

A further major finding was that the age of the overall top ten finishers and the ten fastest swimmers, the ten fastest cyclists, and the ten fastest runners increased across time for both women and men. Similar findings have been reported for athletes competing in Triple Iron ultra-triathlons and Deca Iron ultra-triathlons where the age of the finishers increased across years [39].

A potential explanation for the top ten athletes becoming older and faster in the same time could be the aspect of experience. In Ironman triathletes, the personal best time in an Olympic distance triathlon is a strong predictor variable for Ironman race time [36,40-42]. In ultra-triathletes competing in distances longer than an Ironman such as a Triple Iron ultra-triathlon, the personal best time in an Ironman triathlon and in a Triple Iron triathlon were positively and highly significantly related to overall race time [27]. In ultra-triathletes finishing a Deca Iron ultra-triathlon, both the number of finished Triple Iron ultra-triathlons and the personal best time in a Triple Iron ultra-triathlon were related to overall race time [43]. The number of Olympic distance triathlons worldwide is high; however, the number of Ironman triathlons worldwide is low (http://ironman.com). Since Ironman triathlons as a means to gain experience in long-distance triathlons are little available, the number of experienced athletes in distances of longer than an Ironman triathlon will become dramatically reduced. While in 2012, 1,984 athletes started in Ironman Hawaii and 1,886 starters (95%) finished; a total of 1,686 athletes started in a Double Iron ultra-triathlon between 1985 and 2009 [9]. Of these 1,686 starters, 1,333 athletes finished (79.1%).

Apart from this aspect, recent studies showed that the performance in master Ironman triathletes improved [13,24]. In ‘Ironman Switzerland’, a qualifier for Ironman Hawaii, the number of master triathletes increased, and their performance improved [24]. In Ironman Hawaii, men older than 44 years and women older than 40 years improved both split times and overall race times [13]. Since in both Ironman Switzerland [24] and Ironman Hawaii [13], the participation in master triathletes increased, and these master triathletes improved their performance across years; this might explain that also the top ten athletes may become older across years but still seem to be able to improve their performance.

### Limitations

This study is limited since potential predictor variables for an ultra-endurance performance such as body composition [44], previous experience [26], training intensity [44], nutrition [45,46], motivation [46,47], and weather conditions [14,48] were not considered. These variables might have influenced race outcome.

## Conclusion

To summarize, for overall top ten finishers, the sex difference decreased across years for overall race time and running, but not for swimming and cycling. For the top ten per discipline, the sex difference in performance remained unchanged. The athletes improved their performances across years although the age of peak performance increased. Future studies need to investigate the background of elite Ironman triathletes whether they came from swimming, cycling, or running.

## Competing interests

The authors declare that they have no competing interests.

## Authors’ contributions

CAR wrote the manuscript, BK collected the data, RL performed the statistical analyses, and TR participated in the design and coordination and helped draft the manuscript. All authors read and approved the final manuscript.
